# Toxicity drives facilitation between 4 bacterial species

**DOI:** 10.1073/pnas.1906172116

**Published:** 2019-07-03

**Authors:** Philippe Piccardi, Björn Vessman, Sara Mitri

**Affiliations:** ^a^Department of Fundamental Microbiology, University of Lausanne, CH-1015 Lausanne, Switzerland;; ^b^Swiss Institute of Bioinformatics, CH-1015 Lausanne, Switzerland

**Keywords:** cooperation, competition, stress gradient hypothesis, species diversity, community function

## Abstract

Competition between microbes is extremely common, with many investing in mechanisms to harm other strains and species. Yet positive interactions between species have also been documented. What makes species help or harm each other is currently unclear. Here, we studied the interactions between 4 bacterial species capable of degrading metal working fluids (MWF), an industrial coolant and lubricant, which contains growth substrates as well as toxic biocides. We were surprised to find only positive or neutral interactions between the 4 species. Using mathematical modeling and further experiments, we show that positive interactions in this community were likely due to the toxicity of MWF, whereby each species’ detoxification benefited the others by facilitating their survival, such that they could grow and degrade MWF better when together. The addition of nutrients, the reduction of toxicity, or the addition of more species instead resulted in competitive behavior. Our work provides support to the stress gradient hypothesis by showing how harsh, toxic environments can strongly favor facilitation between microbial species and mask underlying competitive interactions.

## Significance

Microbial communities play a major role in our lives, but we understand little about how species within them interact. Here, we studied 4 bacterial species that could degrade toxic industrial fluids. We expected these species to compete, but instead found that they all benefited from each other: Alone, only 1 species could survive, while together they all grew and degraded the fluid. However, this result depended on the environment. Positive interactions were most common in the toxic fluid, and, if we made survival easier, for example by adding nutrients, bacteria began to compete. Our findings provide a simple intuition: In a harsh environment where single species are unable to grow, the only option becomes to work together.

A microbial cell living in the human gut, in the soil, or in a biofuel cell is typically surrounded by cells of its own kind as well as other strains and species. The way in which it interacts with other community members is key to its growth and survival, and, ultimately, to the stability and functioning of the community as a whole. Being able to predict community dynamics and functioning over ecological and evolutionary time scales is not only fundamentally interesting but can also help develop therapies for microbiome dysbiosis or augment soil to improve agricultural productivity (1–4).

A central question in studying microbial interactions is whether community members cooperate or compete with one another (5–7). Stable cooperation that evolves in 2 interacting species because of their benefit to one another (6) is only expected under highly restrictive conditions (8, 9), with few documented examples (10). Facilitation (11) is more prevalent, since it encompasses cooperation as well as commensalism, where one species accidentally benefits from another, for example by cross-feeding off its waste products (12–15). It appears, however, that microbial life is mostly competitive: Microbes have evolved a great number of ways to harm other strains and species, which gives them a competitive advantage for available resources, be they nutrients, oxygen, or space (16). Our base expectation is therefore that microbial species will tend to compete (6, 8).

However, whether species help or harm each other is likely to depend on the environment they are in (17–20). The stress gradient hypothesis (SGH) (21) predicts that positive interactions should be more prevalent in stressful environments, while permissive environments should favor competition. The hypothesis has only rarely been tested in microbial communities (17, 20, 22, 23), and the studies that have tested it involve either species whose interactions have been genetically engineered (20), theoretical work (23), or communities containing many species (17, 22), where it is difficult to quantify individual species abundances and their interactions, and to understand why observations are in line with the SGH.

To fill this gap, here we used a synthetic community composed of 4 bacterial species that has been applied to the bioremediation of highly alkaline and polluting liquids used in the manufacturing industry, called metal working fluids (MWF) (24–26). MWFs contain chemical compounds that are rich nutrient sources for bacteria, such as mineral oils and fatty acids (27), as well as biocides that inhibit microbial activity (26). The 4 strains—identified as *Agrobacterium tumefaciens*, *Comamonas testosteroni*, *Microbacterium saperdae*, and *Ochrobactrum anthropi*, and named str. MWF001 (*SI Appendix*, section S1)—were previously isolated from waste MWF and selected based on their ability to individually survive or grow in MWF (25). The synthetic community was shown to degrade the polluting compounds in MWF more efficiently and reliably than a random community (25, 28). This community, in its defined chemical environment, represents a tractable model system for exploring how abiotic and biotic interactions shape the ecological dynamics of microbial communities. By quantifying MWF degradation efficiency and mapping it to species composition and their interactions, this model system can also help answer another key question in microbial ecology: How do interspecies interactions affect ecosystem functioning?

## Results

### Facilitation Dominates the Community in MWF.

**Fig. 1.**
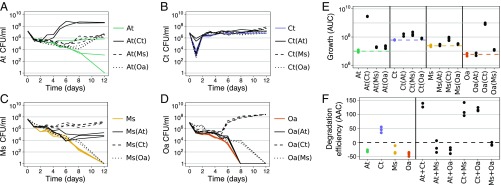
Comparison of mono- and pairwise cocultures. (*A*−*D*) Population size quantified in colony-forming units per milliliter over time for monocultures (in color) and pairwise cocultures (in black; coculture partner indicated in brackets). In the cocultures, each species could be quantified separately by selective plating. Each panel shows the data for 1 species: (*A*) *A. tumefaciens* (At), (*B*) *C. testosteroni* (Ct), (*C*) *M. saperdae* (Ms) and (*D*) *O. anthropi* (Oa). (*E*) AUC in *A*−*D*. Dashed lines indicate the mean of the monocultures, shown in color. Statistical significance and interaction strengths are calculated based on combined data from this and the repetition experiment (*SI Appendix*, Fig. S1), and shown in Fig. 3 and Dataset S1. (*F*) AAC describing the decrease in COD (see *Materials and Methods*) (i.e., degradation efficiency; *SI Appendix*, Fig. S6 *A* and *B*). Negative AAC values arise because dead cells increase the COD (*SI Appendix*, Fig. S7). AUC (*E*) and AAC (*F*) correlate positively (*SI Appendix*, Fig. S4).

We first characterized the effect of each species in the MWF community on the others. The 4 species were incubated alone (monoculture) or in combination with a second species (pairwise coculture) in shaken flasks containing MWF medium over 12 d (see *Materials and Methods*). The inoculum volume for each species was held constant across all conditions, i.e., the total was higher in cocultures. In monoculture, *C. testosteroni* was able to survive and grow in MWF, while *A. tumefaciens* survived in some replicates, and *M. saperdae* and *O. anthropi* did not (Fig. 1 *A*–*D*). Qualitatively similar results were obtained in an independent repeat of the experiment (*SI Appendix*, Fig. S1).

We quantified species interactions by comparing the area under the growth curve (AUC) of monocultures and pairwise cocultures and define an interaction as negative or positive if the AUC of the coculture is significantly smaller or greater than the AUC of the monoculture, and neutral otherwise (see *Materials and Methods*). Defining interactions by the AUC means that they may vary with the length of the experiment and the inoculum volume, but the measure nevertheless combines growth rate, death rate, and final yield in 1 value. Using this measure, positive interactions dominated the MWF ecosystem (Fig. 1*E* and *SI Appendix*, section S1; see Fig. 3*A*). *C. testosteroni* promoted the survival and growth of all other species, while also benefiting significantly from the presence of *A. tumefaciens* and *M. saperdae*. *M. saperdae* and *O. anthropi* also slightly reduced each other’s death rates (Fig. 1 *C* and *D*). Finally, *A. tumefaciens* rescued *M. saperdae* from extinction (Fig. 1*C*), but the AUC was not significantly different from *M. saperdae* in monoculture. These positive interactions were still observed if we kept the inoculum constant between cocultures and monocultures (*SI Appendix*, Fig. S5 and section S4), suggesting that the 4 species functionally complement each other.

Degradation efficiency in all cocultures that included *C. testosteroni* was higher compared with any of the monocultures (Fig. 1*F*). More generally, degradation efficiency correlated positively with population size (*SI Appendix*, Fig. S4; Spearman’s ρ=0.77, $P<10^{-15}$).

We wondered whether these positive interactions were specific to these 4 species, which may have adapted to each other’s presence in the past (28). To test for this, we grew 6 new isolates, that had never previously interacted with our 4 species, in pairwise cocultures with *C. testosteroni* and found that 4 out of 6 could only survive in the presence of *C. testosteroni*, and 3 affected it positively in return (*SI Appendix*, Figs. S2 and S3). This suggests that these positive interactions are likely to be accidental rather than having evolved because of their positive effect (facilitation rather than evolved cooperation).

Together, these first results appear to contradict the expectation that competition should dominate interactions among microbial species (6, 8). However, according to the SGH (21), we expect abiotic stress to induce facilitation. Indeed, since MWF is designed to be sterile, it contains biocides, making it a tough and stressful environment for bacteria (26). We next asked whether the observed positive interactions were due to the toxicity of MWF.

### A Resource-Explicit Model Predicts That Positive Interactions Occur in Toxic Environments.

**Fig. 2.**
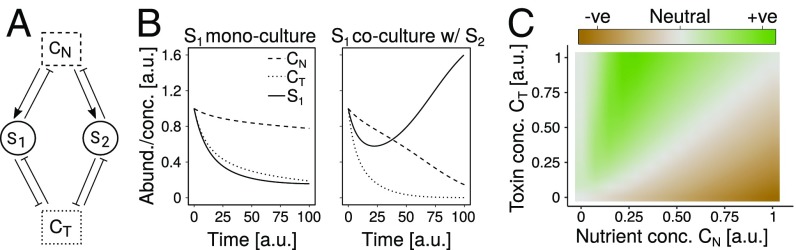
(*A*) In our mathematical model, species $S_{1}$ and $S_{2}$ share a substrate containing nutrients and toxins at concentrations $C_{N}$ and $C_{T}$. The species take up the same nutrients, and invest a fraction of these into toxin degradation and the rest into population growth. Toxins cause cell death and population decline. (*B*) Example results of the model (parameters in *SI Appendix*, Table S3), shown as the abundance of species $S_{1}$ (solid line) and concentrations of nutrients and toxins (dashed and dotted lines, respectively). In monoculture, $S_{1}$ goes extinct due to toxins (*Left*), but survives in coculture with $S_{2}$ (*Right*). (*C*) The response of one species to the presence of another is measured as the difference in AUC between the coculture and monoculture (color and parameters in *SI Appendix*, Table S3) and shown as a function of nutrient and toxin concentrations. At high toxin concentrations and intermediate nutrients, interactions are positive (+ve) due to the joint degradation of toxins (as in *B*). As nutrients are increased or toxins decreased, competition for limited resources dominates (-ve, short for “negative”).

To explore the possibility that interactions were due to toxicity, we constructed a mathematical model that describes interspecies interactions through their common exposure to nutrients and toxins in batch culture (Fig. 2*A*). Our model extends MacArthur’s consumer resource model (29). For simplicity, we initially considered 2 species that share and compete for a single limiting nutrient, and are killed by the same toxin, but do not interact otherwise (see *SI Appendix*, section S1). Species deplete the nutrients as they grow, and can invest a proportion of their growth into degrading the toxin. To match the experiments, we solved the system of equations for each species in monoculture and coculture with a second species and defined (unidirectional) interactions as the difference between the area under the 2 growth curves. We then used the model to ask how interactions vary as a function of initial nutrient and toxin concentrations.

If nutrients are low and toxicity is high, species in the model die out regardless of whether they are in monoculture or coculture (gray area on far left of Fig. 2*C*). As nutrients are increased, the cocultured species manage to degrade the toxins sufficiently, while bacteria in monoculture cannot survive (Fig. 2*B*). In this area of the state space (green area in Fig. 2*C*), the presence of the second species has a positive effect on the first (rescuing it from death) despite the underlying competition for nutrients. As nutrients are further increased, however, growth rates increase and toxins can be degraded sooner, such that the presence of a second species becomes unnecessary and even detrimental to the first. The lower the toxin concentration, the faster this competitive effect arises (Fig. 2*C*). In sum, high toxicity and intermediate nutrients, where species cannot survive alone, is where species in our model benefit from the presence of others. We hypothesized that this regime best describes the 4 species’ growth in MWF.

When the 2 species have the same model parameters, positive interactions rely on the coculture being inoculated with twice as many cells as the monoculture, and hence twice the degradation effort. According to our experiments, however, positive interactions still dominate even if the total cell number at the beginning is constant, suggesting that facilitation occurs because different species degrade different toxins (*SI Appendix*, Fig. S5). To better represent this effect, we extended our model in *SI Appendix*, section S3 by introducing a second toxin, and letting each species degrade 1 of the 2 toxins. In this extended model, as in the experiments, positive interactions arise even when the total cell number is constant.

### The Effect of Environmental Changes on Interactions Matches Model Predictions.

In the model, positive interactions dominate at high toxicity, given that sufficient nutrients are present. Increasing nutrient concentrations further or reducing toxicity instead increases competition. We assumed that our bacteria in the MWF lay at the point in the state space where positive interactions are favored, and modified the environment in 3 additional experiments to test the model’s predictions.

**Fig. 3.**
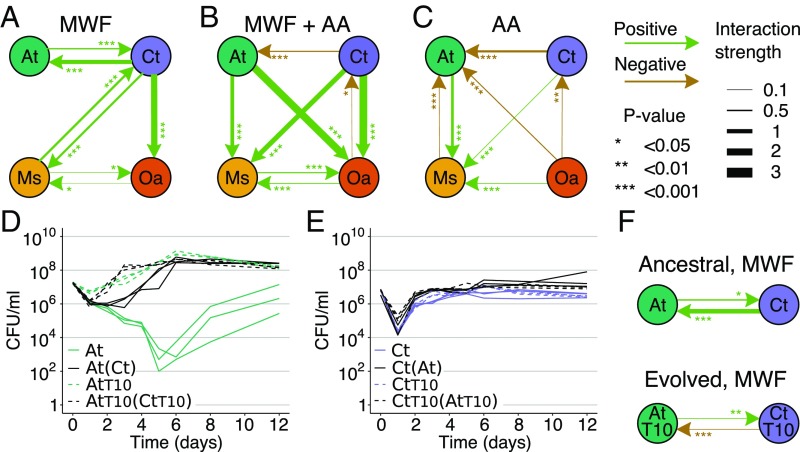
Pairwise interaction networks under different environmental conditions. Positive/negative interactions indicate that the species at the end of an arrow grew significantly better/worse in the presence of the species at the beginning of the arrow in (*A*) MWF, (*B*) MWF + AA, and (*C*) AA medium. Arrow thickness represents interaction strength as the 10-fold change in the coculture AUCs compared with monoculture AUCs, i.e., by how many orders of magnitude a species changed the AUC of another. Statistical significance and interaction strengths were calculated based on 2 experiments in *A* (data in Fig. 1 and *SI Appendix*, Fig. S1), and 1 experiment in *B* (*SI Appendix*, Fig. S9) and *C* (*SI Appendix*, Fig. S8). *P* values and interaction strengths are listed in Dataset S1. (*D*) Monoculture and coculture growth curves of ancestral At and (*E*) Ct versus the same strains evolved in monoculture for 10 wk (AtT10, CtT10). Coculture partners are indicated in brackets. (*F*) Interactions between ancestral and evolved At and Ct strains based on growth curves in *D* and *E*. Arrow widths and asterisks are as defined for *A*–*C*. The interactions between At and Ct in *A* and *F* have different strengths and *P* values because they come from different experimental repeats.

We first increased the concentration of nutrients in the MWF medium by adding 1% Casamino acids (AA) (see *Materials and Methods*), which is a nutrient source for 3 out of the 4 species (*SI Appendix*, Fig. S8). In this supplemented MWF medium (MWF + AA), monocultures of *A. tumefaciens* and *C. testosteroni* immediately grew well, while *M. saperdae* and *O. anthropi* still suffered from its toxicity (*SI Appendix*, Fig. S9). According to the model, we expect competition between the 2 species that could grow. Indeed, the 2-way positive interaction between *C. testosteroni* and *A. tumefaciens* switched to negative in 1 direction (Fig. 3*B*), indicating that a change in nutrient composition can radically modify bacterial interactions. The 2 species that still experienced the environment as toxic (*M. saperdae* and *O. anthropi*) became the only 2 species benefiting from being in pairwise cocultures. They also started to benefit from *A. tumefaciens* and benefited more from *C. testosteroni* that could grow better (and presumably detoxify faster) in this medium than in MWF.

Second, we reduced toxicity by growing the bacteria in 1% AA. Ideally, we would have removed toxic compounds from MWF, but MWF is chemically complex and only sold as a finished product. By removing MWF entirely, the growth medium was no longer toxic, but nutrients were also reduced and may have become differently accessible. Caveats aside, according to the model, we expected negative interactions to increase. Indeed, we found all interactions to be negative, except for *M. saperdae*, whose growth was significantly promoted by all 3 remaining species. *M. saperdae*’s inability to grow in monoculture in AA (*SI Appendix*, Fig. S8*C*) suggests that it relies on cross-feeding from the other 3 species. While our mathematical model does not explicitly capture cross-feeding interactions and assumes that all species compete for the same nutrient, such positive interactions are common in microbial communities (12).

A final way by which we simulated a reduction in environmental toxicity was to allow the bacteria to individually adapt to MWF. We reasoned that, if the species evolved to sustain their own growth in MWF, they would lose their positive effects on one another. To test this hypothesis, we conducted experimental evolution on *A. tumefaciens* and *C. testosteroni* by passaging each species alone in MWF for 10 wk (see *Materials and Methods* and *SI Appendix*, Fig. S10). We did not do this for *M. saperdae* and *O. anthropi* because they could not grow alone in MWF (Fig. 1 *C* and *D*). After 10 wk, *A. tumefaciens* grew significantly better in MWF, suggesting that it evolved to become more tolerant to its toxicity (Fig. 3*D*). In the model, this represents a reduction in toxicity. By again comparing monocultures and cocultures, we found that the positive effect of *C. testosteroni* on *A. tumefaciens* in the ancestral strains switched to competitive in the evolved strains, as predicted by the model (Fig. 3 *D*–*F*).

Taken together, these results show that positive interactions in our system were most common at high levels of abiotic stress and intermediate nutrient concentrations where most species could not grow, while making the environment more habitable promoted competition. This observation is in line with the SGH. We next took advantage of our system to ask how interactions change with increasing community size.

### Interactions between More Than 2 Species Depend on Environmental Toxicity.

**Fig. 4.**
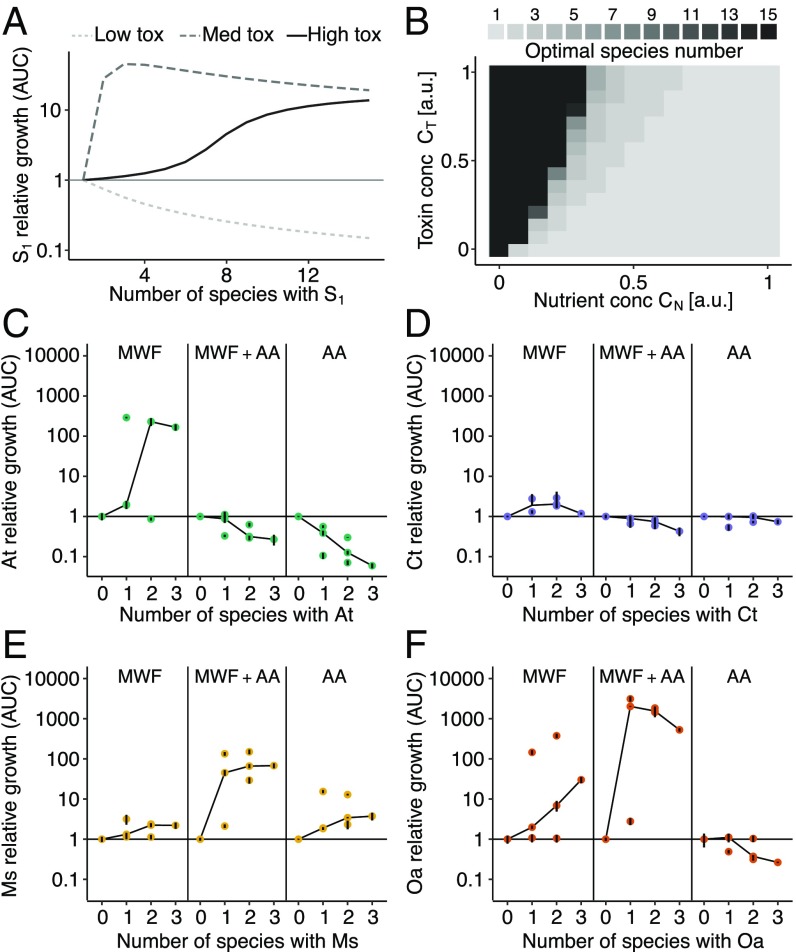
(*A*) Our model predicts that, for a focal strain $S_{1}$, an increasing community size eventually becomes detrimental. The number at which such competition starts depends on environmental toxicity. (*B*) The optimal number of species with respect to the AUC of a focal strain (peak in *A*) varies with nutrient and toxin concentrations. (*C*–*F*) Each species’ growth expressed in fold change in its AUC divided by its mean monoculture AUC in the 3 different media. Each point shows the mean of a culture treatment composed of 1 to 4 species, and vertical black lines show standard deviations. Black lines connect the median points. In environments where a species could not grow alone, the curves are hump-shaped, while, in more benign environments, species grow less well in the presence of others.

Our model predicts how the sign of interactions changes with respect to increasing species numbers: In a benign environment with low toxicity, a focal species should grow worse with increasing species number (competition; Fig. 4*A*). When the number of species is increased in a stressful environment, the increased degradation effort first leads to facilitation. However, when enough (functionally equivalent) species are present to alleviate the stress, competition should begin to dominate once again, leading to a hump-shaped curve (Fig. 4*A*, medium toxicity). This competition arises in the model because all species consume the same nutrient, and would be predicted for species whose niches overlap. The community size at which species benefit most from the presence of others (the optimal number of species) depends on the environment, as shown in Fig. 4*B*.

To test these predictions, we pooled our monoculture and pairwise coculture data (Fig. 1) with experiments where we grew our species in groups of 3 and 4 in all 3 media and calculated the AUC (*SI Appendix*, Figs. S11–S13). In MWF, all species grew better as community size increased (Fig. 4 *C*–*F*, *Left*). However, this benefit leveled off eventually, resulting in hump-shaped or saturating curves. In MWF + AA, only *M. saperdae* and *O. anthropi*, the 2 species that couldn’t grow in this medium alone, showed a hump-shaped curve, while *A. tumefaciens* and *C. testosteroni* grew worse with more species. Finally, in AA, increasing competition was observed for all except *M. saperdae*, which was unable to grow alone (*SI Appendix*, Fig. S8*C*).

In sum, positive interactions occurred in environments that were highly stressful for a species when alone. As this stress was reduced either through the presence of other detoxifying species or due to increased nutrients or decreased toxicity, competitive interactions between them became salient.

### Degradation Efficiency Only Correlates with Species Number in Toxic Environments.

**Fig. 5.**
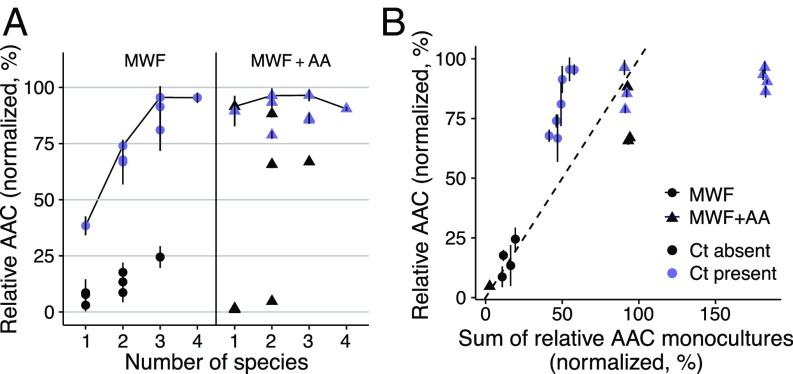
Degradation efficiency as a function of species number. (*A*) AAC of COD (see *Materials and Methods*), normalized to values between 0 and 100%. Points show the mean of a culture treatment composed of 1 to 4 species, and vertical lines show standard deviations. Blue (or black) points show cultures where *C. testosteroni* was present (or absent). Cultures growing on MWF (*Left*) only reach their maximum degradation potential once 3 species are present (see black line connecting the maximum mean values). In MWF + AA (*Right*), even single species can degrade as efficiently as the best cultures. In a more benign environment, there is less need for a diverse community. (*B*) Prediction of an additive model of the sum of degradation efficiencies of individual species is plotted against degradation efficiency of the cocultures in both growth media. Data points are identical to >1 species in *A*. In MWF, cocultures are more efficient than the sum of the corresponding monocultures (most points above dashed line), while, in MWF + AA, they are equally or less efficient (most triangles below the dashed line). The presence of *C. testosteroni* explains much of the AAC in *A* and *B*.

Finally, we asked how the size of a community affects its degradation ability and whether that depends on the interactions between its members. In MWF, where interactions were positive (Figs. 3*A* and 4 *C*–*F*), increasing species led to better degradation, but did not improve significantly once 3 species were present (Fig. 5*A*; F test comparing the 3-species community with the highest mean area above the curves (AAC) to the AAC of the 4-species community, P=0.96). Instead, in MWF + AA, where *A. tumefaciens* and *C. testosteroni* experienced competition when other species were added (Fig. 4 *C* and *D*), degradation efficiency already reached its maximum with a single species, and did not significantly improve in a larger community (P=0.74 for F test comparing AACs of the communities with the highest mean AAC for each community size). Regardless of whether we added AA to the medium, however, a similar final amount of undegraded medium remained in the 4-species communities (*SI Appendix*, Fig. S15). Interestingly, the total population size already saturated at 2 species in MWF (*SI Appendix*, Fig. S16), suggesting that the benefit in degradation efficiency of a third species is not only due to a larger population size but to functional complementarity.

The contrast between the 2 media becomes even clearer if we apply an additive null model to degradation efficiency (i.e., degradation of each species is independent of the other): Does the sum of monoculture degradation efficiencies predict that of the corresponding coculture? In line with the observed interactions, cocultures growing in MWF degraded better than the sum of their monocultures, while, if amino acids were added, the benefit of additional species became minimal (Fig. 5*B*). A similar analysis on 72 strains (8) found that only a few species pairs were more productive in coculture relative to the prediction of an additive model. Using the same model here, we show that community functioning in coculture (i.e., degradation efficiency) changed from being greater to smaller than the null model prediction by simply changing nutrient concentrations.

## Discussion

In our model system, facilitative interactions between species occurred in a toxic environment, where only a few community members could survive. By presumably improving the environment for their own survival, these species may have accidentally allowed each other to thrive. Once conditions were sufficiently benign, however, competition dominated. These data provide an intuitive explanation for the SGH.

Based on our results, we predict that, in toxic environments, species can coexist even if they compete for a single resource, as long as a subset of them participates in detoxification, and the level of toxicity is low enough for at least 1 detoxifier to survive. Coexistence between species can, of course, be promoted by other processes, including resource partitioning, spatial and temporal heterogeneity, dispersal, and cross-feeding (30). Recently, Goldford et al. (12) showed that species competing for a single carbon source can coexist through niche creation: secreting metabolic by-products that others use to grow. The growth of *M. saperdae* here may depend on such cross-feeding (Figs. 3*C* and 4*E*). The remaining facilitative interactions in our study instead likely arose by species removing toxic compounds to enable others to grow and access niches for which they compete. Similar dynamics are expected for antibiotic-degrading bacteria in environments containing antibiotics (31). Indeed, antibiotic degraders can protect neighboring cells from antibiotics (32–34).

One important caveat is that we do not know the molecular mechanisms behind the interactions in our system or the process of MWF degradation. These may be important for predicting its behavior. For example, whether degradation occurs through the passive uptake of toxins or through costly enzyme secretion will alter predictions on evolutionary stability. It is also unclear why *C. testosteroni*’s population dropped drastically before exponential growth (Fig. 1*B*). Our model assumes that cells start to grow when enough toxins have been degraded, but it may instead have been because of slow changes in gene expression patterns. Finally, we cannot be sure that facilitation occurs through toxin degradation. However, the positive effect of *C. testosteroni* on many other species (Fig. 3*A* and *SI Appendix*, Fig. S2) suggests that it is toxin removal rather than metabolite secretion that so many different species are benefiting from.

Nevertheless, our data help address our original question: What makes species in microbial communities help or harm each other? In all of the environments where our species could grow, they competed with one another, suggesting that competition is the underlying dynamic between them. Positive effects were, instead, only observed when species were unable to survive or grow alone. Whether to describe these interactions as cooperative is debatable. A conservative, evolutionary definition of cooperation requires that the relevant phenotype is selected for because of its positive effect on other species (5, 6, 8, 10). Since we have no information on the evolutionary history of the observed behavior, we prefer to refer to it as facilitation (11, 35) and assume that the interactions are an accidental side effect of each species detoxifying the MWF for its own benefit.

Another major debate in current ecology is on the importance of higher-order interactions (HOIs) (36–40). While we do not explicitly study HOIs here, we provide a logical argument as to why they may be unavoidable: Since each new species added to a community is likely to modify the concentrations of nutrients and toxins, and we know that these concentrations can alter interactions between species pairs (Fig. 2C), then new species can surely modify existing interactions as described by phenomenological models (39). Our argument highlights the need for more mechanistic, resource-explicit models in ecology (41, 42). Models with context-dependent interactions would also allow one to carefully engineer the environment to manipulate community dynamics (43).

In engineering synthetic microbial communities for practical applications (2, 4, 44), it has been observed that community function saturates with increasing species diversity (45, 46). Here, the rate at which MWF degradation efficiency saturated depended on environmental toxicity (Fig. 5). This suggests that a harsh environment might require a larger community whose members can facilitate each other’s growth to achieve the desired task. In contrast, making the environment too permissive can reduce the potential benefits of increasing community size due to competition arising between its members. Designing stable consortia in environments where many species are able to grow may therefore be difficult.

Disentangling interactions between species and their effect on community function remains challenging (1), but can be approached using accessible model systems such as this one that use natural bacterial isolates. With this approach, we aim to develop a fundamental understanding that can later be extended to the complexity of natural microbial communities.

## Materials and Methods

Detailed methods are described in *SI Appendix*, section S1. The 4 bacterial strains used in the main study were isolated from waste MWF (25, 47), which is less toxic than the fresh MWF we are preparing here. Additional species kindly donated by Peter Küenzi from Blaser Swisslube AG, Hasle-Rüegsau, Switzerland, are listed in *SI Appendix*, section S1.

Species were first grown alone in tryptic soy broth (TSB) overnight, diluted to an optical density at a wavelength of 600 nm of 0.05 and grown for 3 h in TSB to obtain ∼$10^{6}\text{to}10^{7}$ CFU/mL at the beginning of each experiment. For each species, 200 μL of these cultures were harvested (e.g., 400 μL for pairwise cocultures), washed, and resuspended in 30 mL of 1 of 3 media: 1) Castrol Hysol^TM^ XF MWF at a concentration of 0.5%, diluted in water, salts, and metal traces; 2) MWF medium supplemented with 1% AA (MWF + AA); and 3) salts and metal traces supplemented with 1% AA (*SI Appendix*, Table S1). Each treatment was grown in triplicate at $28{}^{○}$C, 200 rpm for 12 d, together with a sterile control. On days 1 to 6, 8, and 12, populations were quantified by serial dilution and plating (colony-forming units) and distinguished by selective plating on antibiotic plates. The main experiment was repeated twice, and we used a blocked ANOVA with “experiment” as a random effect to test for significant interactions. Other experiments were performed once, and F tests were used (*P* values in Dataset S1). Degradation efficiency was measured by comparing the decrease in chemical oxygen demand (COD), a proxy for the total carbon, in the cultures and a sterile control over time (AAC). COD was measured using Macherey Nagel 15 g/L COD tube tests.

To adapt *A. tumefaciens* and *C. testosteroni* to MWF, they were grown alone for 7 d, 300 μL of this culture was transferred into 30 mL of fresh MWF medium, and the procedure was repeated for 10 wk (*SI Appendix*, Fig. S10). One colony was then isolated from the first replicate of the evolved populations of *A. tumefaciens* and *C. testosteroni*, and the interactions between them were quantified.

The resource-explicit mathematical model is described in *SI Appendix*, section S1. All data used to generate figures are available in Dataset S2.

## Supplementary Material

Supplementary File

Supplementary File

Supplementary File

Supplementary File
