# Supplementary material for: Toxicity drives facilitation between 4 bacterial species
Source: Proc Natl Acad Sci U S A. 2019 Jul 3;116(32):15979–84. doi: 10.1073/pnas.1906172116 (PMC6690002; doi:10.1073/pnas.1906172116)
Supplement: Supplementary File [file pnas.1906172116.sapp.pdf]

Supplementary Information (SI)

Supplementary Note S1: Supplementary Methods

Bacterial species and growth media. This study included four bacterial strains, which we refer to as: *Agrobacterium tumefaciens* str. MWF001, *Comamonas testosteroni* str. MWF001, *Microbacterium saperdae* str. MWF001 and *Ochrobactrum anthropi* str. MWF001. *A. tumefaciens* was modified with a Tn7 transposon containing a GFP marker, and *O. anthropi* with a Tn5 transposon containing an mCherry marker (thanks to Semhar Ghebrehiwet Tekle) to allow us to distinguish colonies of all four species (see below). The four bacterial species were isolated from waste MWF in a previous study, based on their ability to degrade different MWF substrates (1, 2). It should be noted the waste MWF is less toxic than the fresh MWF that we are preparing here. The identities of the four species were confirmed through 16S gene sequencing (thanks to Marc Garcia-Garcerà and Bastien Vallat). Six additional species isolated from MWF and kindly donated by Peter Küenzi from Blaser Swisslube AG, were also used for supplementary experiments: *Aeromonas caviae*, *Delftia acidovorans*, *Empedobacter falsenii*, *Klebsiella pneumoniae*, *Shewanella putrefaciens*, *Vagococcus fluvialis*. The species were identified at Blaser Swisslube AG by MALDI-TOF, and confirmed by PCR amplification and 16S gene sequencing (thanks to Marc Garcia-Garcerà and Bastien Vallat).

The Metal-Working Fluid (MWF) used in this study (Castrol Hysol™ XF, acquired in 2016) was chosen because of the ability of the four-species co-culture to grow in it and degrade it. The MWF medium was prepared at a concentration of 0.5% (v/v), diluted in water with the addition of selected salts and metal traces to support bacterial growth (Table S1). In addition to (i) the MWF medium, we also conducted growth experiments in (ii) the MWF medium supplemented with 1% Casamino Acids (Difco, UK) (MWF+AA) and (iii) the same selected salts and metal traces supplemented with 1% Casamino Acids only (AA). This third medium was identical to the second, except for the lack of MWF. All medium compositions are listed in Table S1.

Experimental setup. Before each experiment, each of the four species was independently grown in tryptic soy broth (TSB) overnight starting from a single colony (28°C, 200 rpm) in Erlenmeyer flasks (50 ml) containing 10ml of TSB. The next day, the optical density (OD_{600}) of the overnight cultures was measured using a spectrophotometer (Ultrospec 10, Amersham Biosciences), and each species was then inoculated at a standardized OD_{600} of 0.05 into an Erlenmeyer flask (100 ml) containing 20ml of TSB and grown for 3 hours (28°C, 200 rpm) to obtain bacteria in exponential phase with a final concentration of approximately 10^6-10^7 CFU/ml at the beginning of each experiment. These starting population sizes were quantified through plating on agar (see below).

For mono-cultures, 200μl of this final TSB culture were harvested for each species and spun down at 10,000 rcf for 5 minutes. For co-cultures, 200μl of the TSB cultures of each species were first mixed together (e.g. for 2 species, the total was 400μl), then spun down. Experiments were also conducted where the total was fixed to 200μl (Fig. S5). The supernatant was discarded and the pellet resuspended in 30ml of growth medium (e.g. MWF medium) in 100ml glass tubes. In most experiments, 15 treatments (mono-cultures, all pairwise, triplet and quadruplet co-cultures) were conducted simultaneously in triplicate to give 45 experimental cultures in addition to a sterile control. All tubes were incubated at 28°C and shaken at 200 rpm for a total of 12 days.

Quantifying population size. To quantify the population size of each species over time, 200μl were collected on days 1-6, 8, and 12, from each culture tube, serially diluted and plated onto lysogeny broth (LB) agar or trypticase soy agar (TSA) (Difco, UK) plates and incubated at 28°C to count colony-forming units (CFUs). *C. testosteroni* colonies were visible after 24 hours on TSA, while *A. tumefaciens*, *M. saperdae* and *O. anthropi* were visible after 48 hours on LB agar. To distinguish the latter three species when growing in co-culture, in addition to LB agar, cells were also plated onto LB agar plates containing either: (i) 14.25μg/ml of sulfamethoxazole and 0.75μg/ml of trimethoprim to count only *A. tumefaciens* CFUs; (ii) 2μg/ml of imipenem to count only *M. saperdae* CFUs; or (iii) 10μg/ml of colistin to count only *O. anthropi* CFUs. The fluorescent markers further helped to verify our counts on LB agar.

Quantifying interspecies interactions. To infer interactions between species, we calculated the area under the growth curve (AUC) of each species in mono-culture and in its pairwise co-culture with each of the other 3 species. We repeated the experiment in the MWF medium on two independent occasions, each in triplicate. We used a blocked ANOVA with "experiment" as a random effect to test for significant differences. If the AUC was significantly greater or smaller in a pairwise co-culture ($P<0.05$), we deemed the interaction to be positive or negative, respectively. Calculated P-values are shown in Table S2. For the other two media (MWF+AA and AA) and the evolved strains, the pairwise co-culture experiments were performed once only, so F-tests were used to calculate which interactions were significant.

We further quantified interaction strengths by calculating the fold-change in AUC in the presence of a partner species. Interaction strength is represented graphically through the thickness of the arrows connecting two species. Arrow thickness is proportional to: $\log_{10}$(AUC(co-culture)/AUC(mono-culture)).

Quantifying degradation efficiency (Chemical Oxygen Demand). Chemical oxygen demand (COD) was used as a proxy for the total carbon in the MWF. A significant reduction in COD relative to the sterile control was considered as degradation

and the Area Above the Curve (AAC, the integral between the control and the biotic curve) represents degradation efficiency. Briefly, 1ml of MWF emulsion was harvested at the beginning of the experiment, and on days 1-6, 8, and 12, centrifuged (16,000 rcf for 15 minutes) to remove suspended cells (we found that cellular material increases the COD, Fig. S7). Centrifugation separated the MWF into two liquid phases. The top phase was carefully pipetted and discarded, while 200 μl of the second phase was added to NANOCOLOR COD tube tests, detection range 1-15 g/l by Macherey-Nagel (ref: 985 038), heated at 160°C for 30 mins, cooled to room temperature, and the color change quantified on a LASA 7 100 colorimeter (Hach Lange, UK). This protocol was developed with the help of Guillermo Osuna.

Adapting bacteria to MWF medium. *A. tumefaciens* and *C. testosteroni* were grown in MWF medium as described above for 7 days (28°C, 200 rpm) in five replicate mono-cultures. After 7 days, 30 ml of fresh MWF medium was prepared and 300 μl of the week-old culture transferred into it. This was repeated every week for a total of 10 weeks. At the beginning and at the end of every week, population sizes were quantified using CFUs as described above. After 3 weeks, three replicate populations of *A. tumefaciens* had gone extinct (Fig. S10). After 10 weeks, one colony was isolated from the first replicate of the evolved populations of *A. tumefaciens* and *C. testosteroni*, and the interactions between them quantified.

Resource-explicit mathematical model. We consider a community of n distinct species, where the change in abundance S_i of species i is determined by a growth function ρ_i and mortality μ_i which depend on the concentrations C_N and C_T of the nutrient and toxin as shown in Fig. 2A. Nutrients decrease as a function of the species' growth via the biomass yield Y_i, while toxins decrease according to the species' production rate δ_i of enzymes that degrade the toxin as well as a passive uptake rate κ_i. A fraction f_i of the collected nutrients are invested into active degradation and the rest into growth. This results in the following set of differential equations:

$$\frac{dS_i}{dt} = ((1-f_i)\rho_i(C_N) - \mu_i(C_T))S_i \tag{S1a}$$

$$\frac{dC_N}{dt} = -\sum_{i=1}^{n} \frac{1}{Y_i}\rho_i(C_N)S_i \tag{S1b}$$

$$\frac{dC_T}{dt} = -C_T\sum_{i=1}^{n}(f_i\delta_i\rho_i(C_N) + \kappa_i)S_i \tag{S1c}$$

We assume that the growth and death rates saturate with increasing nutrient or toxin concentrations as:

$$\rho_i(C_N) = r_{\max,i}\frac{C_N}{C_N + K_N} \tag{S2a}$$

$$\mu_i(C_T) = m_{\max,i}\frac{C_T}{C_T + K_T} \tag{S2b}$$

for the nutrient and toxin with half-saturation concentrations K_N, K_T and maximum growth or death rate $r_{\max}$, $m_{\max}$. We implemented the model in Python v3.6 using the SciPy library v1.0 and solved with standard ODE solvers for a set of parameters and initial conditions as listed in Table S3. Fig. S17 shows how changes in these parameters and initial conditions affect the outcome of the model. To generate the heat plot in Fig. 2C, we calculated the difference in the AUC of the simulated time-series, between a simulation with initial abundance $S_1 = S_2 = 1$ and another with initial abundance $S_1 = 1$ and $S_2 = 0$.

References.

1. Christopher J van der Gast and Ian P Thompson. Effects of pH amendment on metal working fluid wastewater biological treatment using a defined bacterial consortium. *Biotechnology and Bioengineering*, 89(3):357–66, 2 2005. ISSN 0006-3592. doi: 10.1002/bit.20351.
2. Christopher J van der Gast and Ian P Thompson. Patent US 8,703,475 B2, 2014.

Supplementary Note S2: Supplementary Figures

Data used to generate these figures is available in Dataset S3.

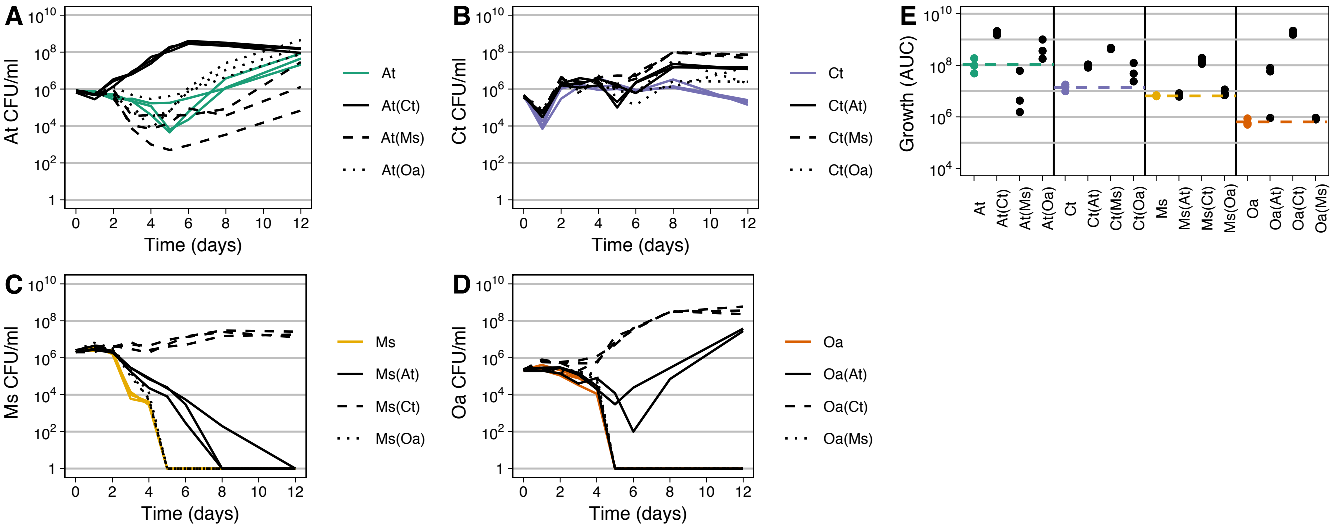

Fig. S1. Repetition of the experiment shown in Fig. 1. At this point in time our protocol for measuring the COD was not yet optimized, so we do not show those data. The AUC data are combined with the data in Fig. 1E to perform the statistical tests shown in Fig. 3A.

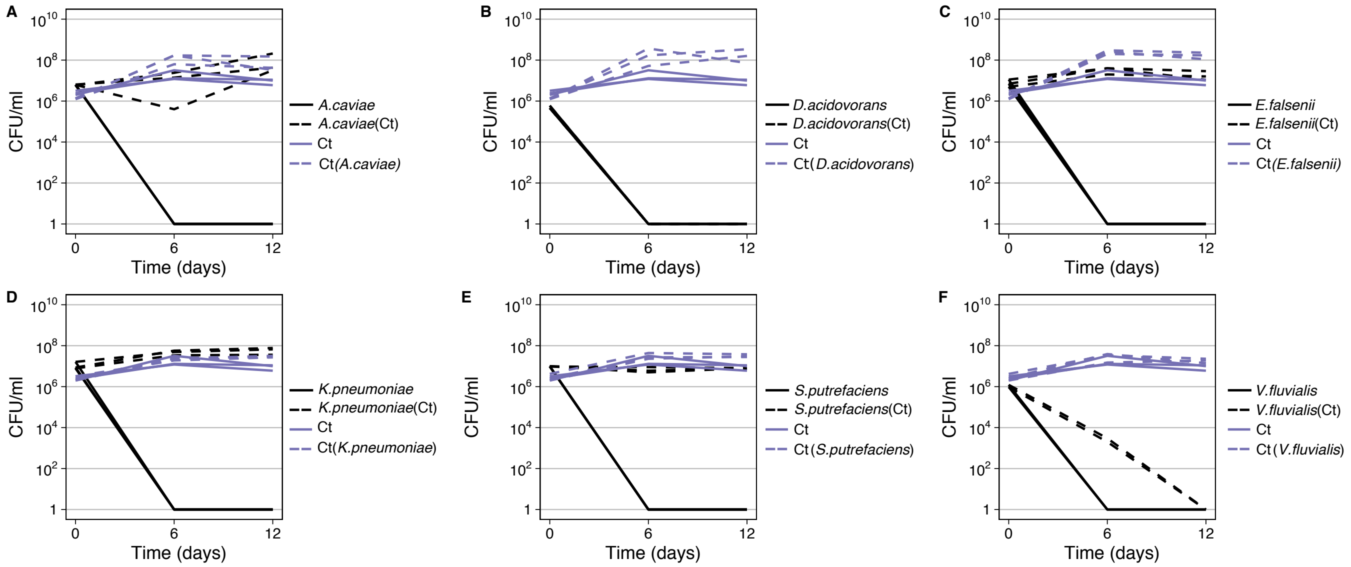

Fig. S2. Pairwise co-cultures in triplicate of *C. testosteroni* (Ct) together with six other strains that were similarly isolated from MWF, but had presumably never interacted previously with *C. testosteroni*. In co-cultures, the first strain mentioned in the label is the one plotted, while the partner strain, if any, is in brackets (e.g. Ct(*A. caviae*) is the growth curve of Ct in the presence of *A. caviae*). All six strains quickly died in MWF if cultured alone, but in the presence of *C. testosteroni*, four out of the six grew better, as shown in Fig. S3. This suggests that the positive interactions at least between *C. testosteroni* and the other three strains shown in Fig. 3 are unlikely to be a product of their co-evolution, but are most likely accidental. Species identities are: (A) *Aeromonas caviae*, (B) *Delftia acidovorans*, (C) *Empedobacter falsenii*, (D) *Klebsiella pneumoniae*, (E) *Shewanella putrefaciens*, (F) *Vagococcus fluvialis*. Sabrina Riveira helped PP collect these data.

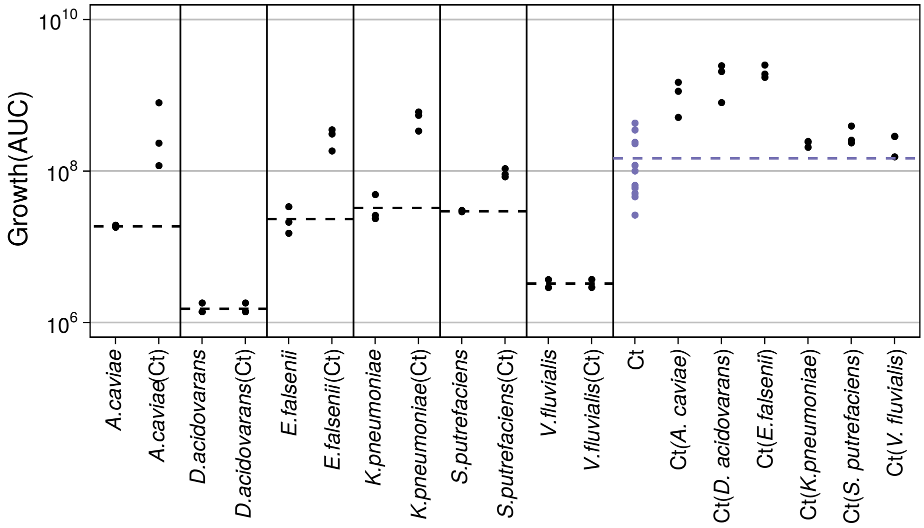

Fig. S3. AUC calculated based on the data in Fig. S2. Four out of the six strains had a higher AUC when grown together with *C. testosteroni*, while *C. testosteroni* also improved its growth in some cases. Note that a mono-culture of *C. testosteroni* was not included every time, but a number of repeats of its AUC are pooled together. This makes a statistical comparison for Ct more difficult, but supports our overall conclusion that positive interactions are likely accidental.

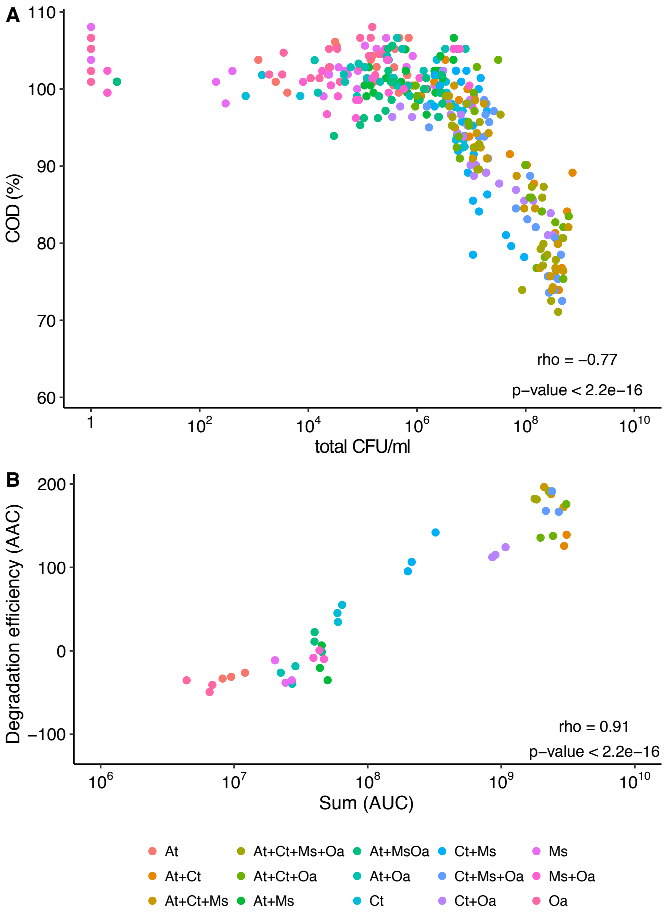

Fig. S4. (A) CFU/ml and COD correlate across experimental conditions (Spearman's correlation test). Data from time-point 0 were not included in this analysis. (B) The sum of growth data (AUC) (e.g. for At+Ct, the AUC of At(Ct) was summed with that of Ct(At) in Fig. 1E) also correlates with degradation efficiency (AAC) (Spearman's correlation test).

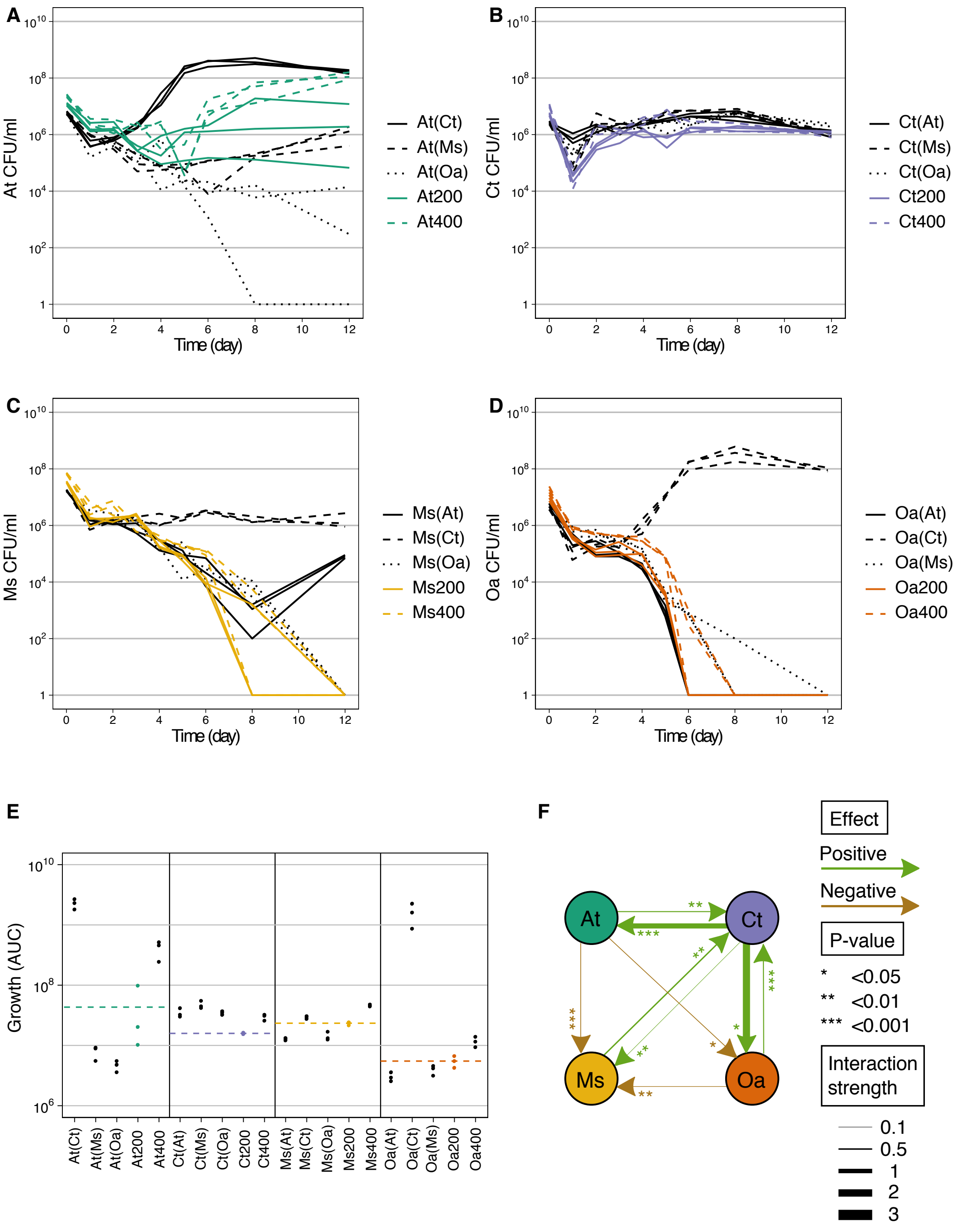

Fig. S5. Repeat of the mono-culture and pairwise co-cultures presented in the main text (Fig. 1) but where co-cultures contained the same total inoculum volume as the mono-cultures (e.g. At200 was inoculated with 200μl of At, while At(Ct) was inoculated with 100μl of At and 100μl of Ct). We also included a treatment where we doubled the volume of the inoculum for the mono-cultures (e.g. At400). (A)-(D) Growth curves for each species, (E) AUC for each treatment and species, (F) interaction network comparing mono-cultures and co-cultures of the same total inoculum volume (e.g. comparing At200 with At(Ct) of 100 each). Arrow width and color is as described for Fig. 3. For all species, doubling the inoculum volume of the mono-culture resulted in AUCs that were significantly larger (all four $P < 0.015$).

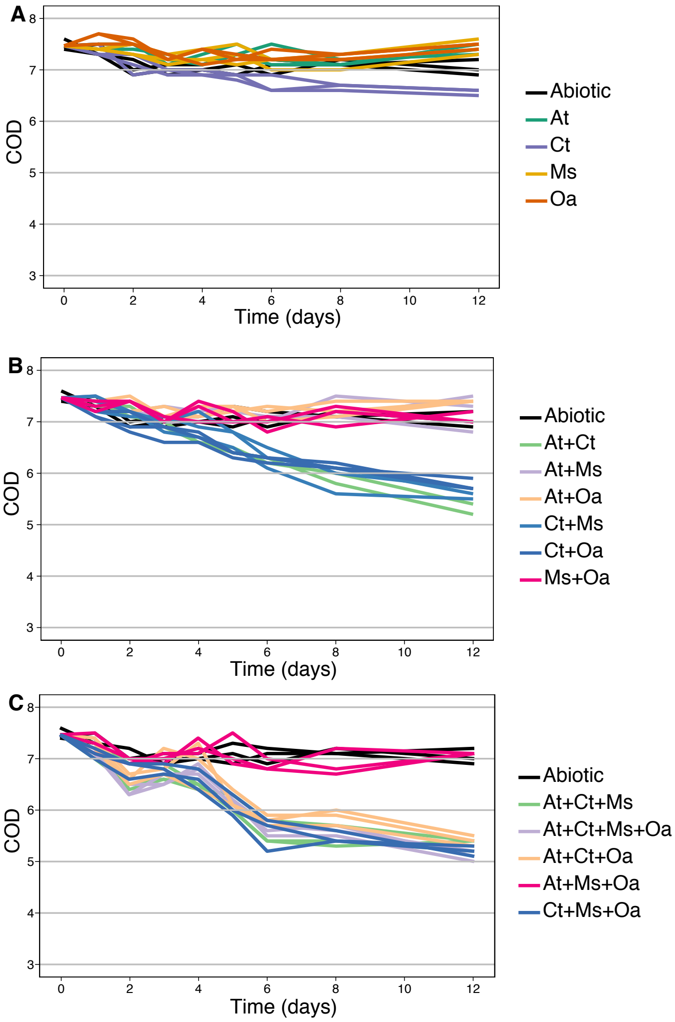

Fig. S6. Chemical Oxygen Demand (COD) over time in MWF medium in (A) mono-cultures, (B) pairwise co-cultures and (C) in three- and four-species co-cultures. Each treatment was performed in triplicate. Black lines show an abiotic control treatment that was sterile. Note that these absolute COD values are not directly comparable to those in Fig. S14, since for each experiment the values in the abiotic control differ somewhat.

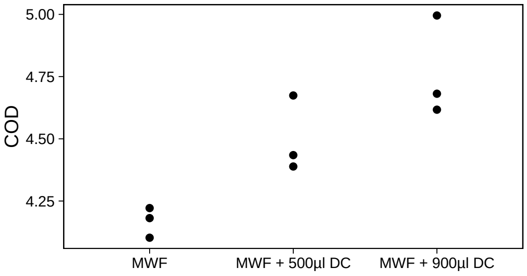

Fig. S7. Chemical Oxygen Demand (COD) of an abiotic control treatment that was sterile compared to two treatments containing increasing concentrations of dead cells. Cells were killed by autoclaving, thanks to Avery Becker. This shows that dead cells can increase the COD.

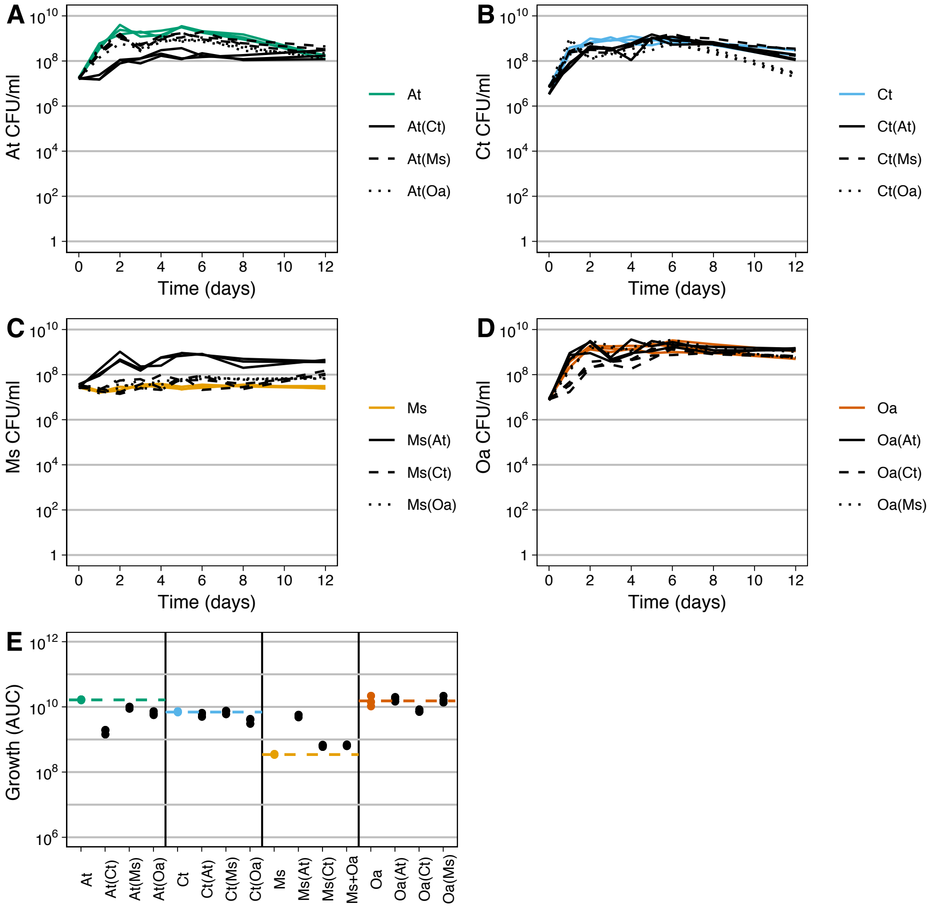

Fig. S8. Growth and pairwise interactions in amino acid medium (AA). Legend explanations are as in Fig. 1.

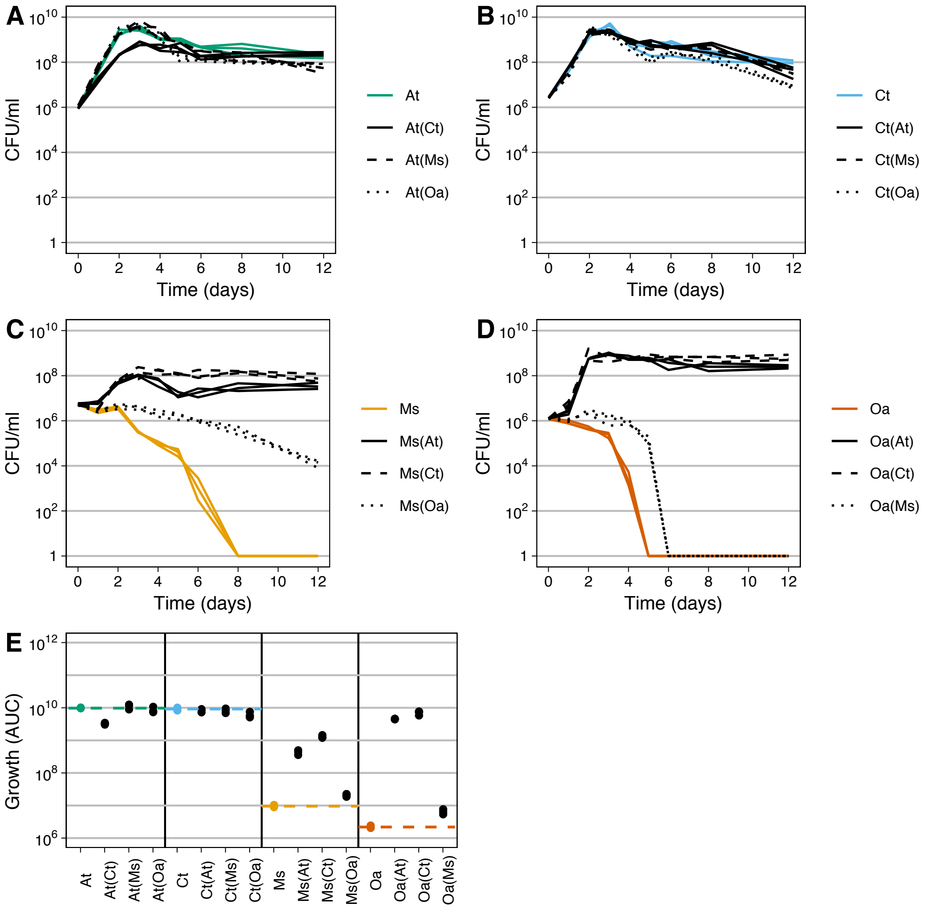

Fig. S9. Growth and pairwise interactions in MWF supplemented with amino acids (MWF+AA). Legend explanations are as in Fig. 1.

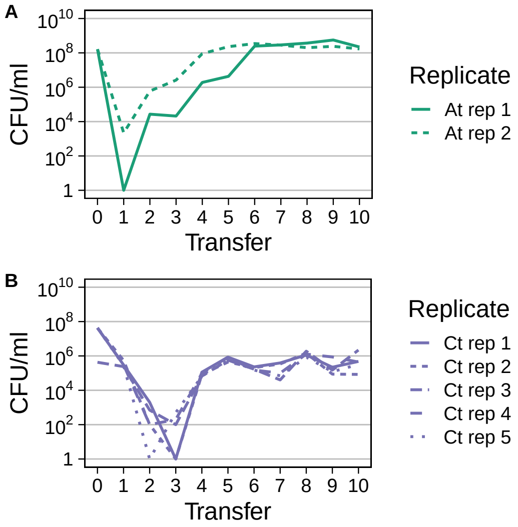

Fig. S10. Evolutionary experiment where mono-cultures of (A) *A. tumefaciens* and (B) *C. testosteroni* were transferred into fresh tubes every week. The plot shows CFU/ml at the end of each week prior to a 1:100 dilution. Five replicates were used for each condition, but in three *A. tumefaciens* cultures, cells went extinct after a few weeks. Clones were taken for each species from replicate one (solid line) for the experiments shown in Fig. 3.

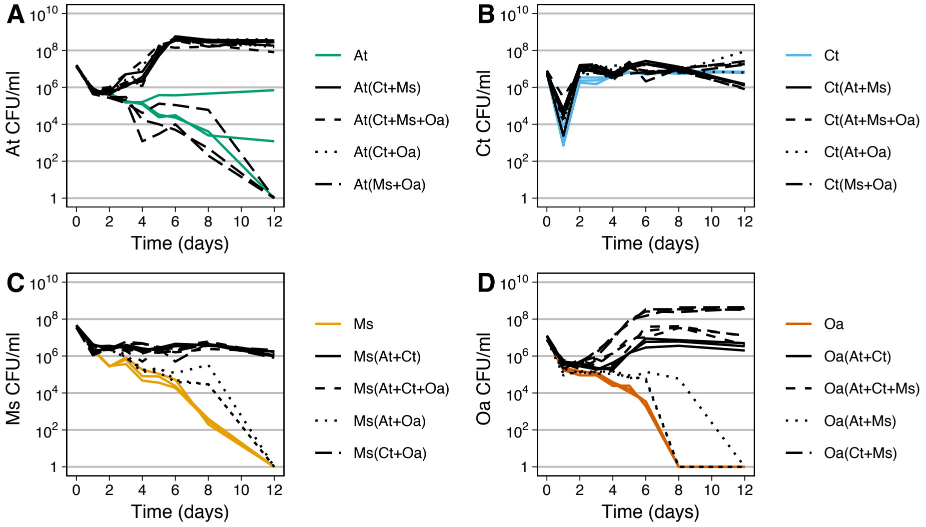

Fig. S11. Growth of three and four-species co-cultures in MWF medium. Legend explanations are as in Fig. 1.

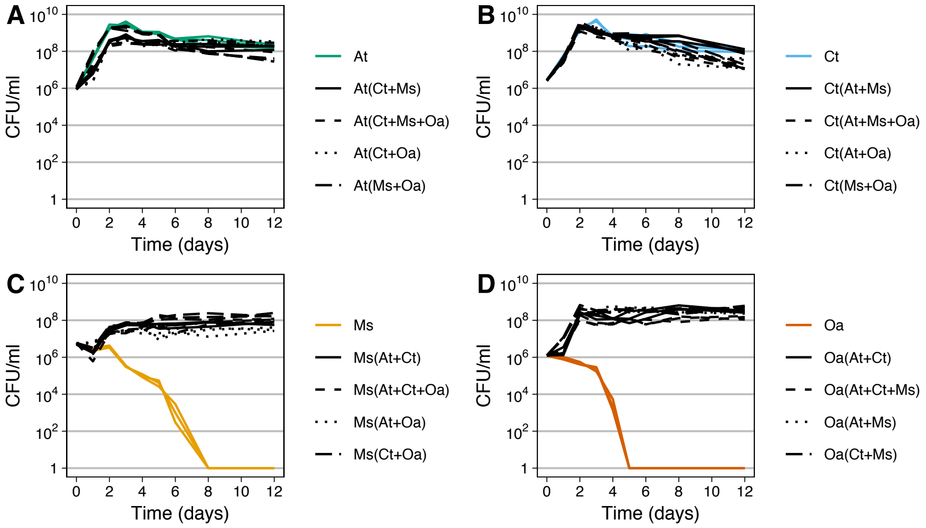

Fig. S12. Growth of three and four-species co-cultures in MWF+AA medium. Legend explanations are as in Fig. 1.

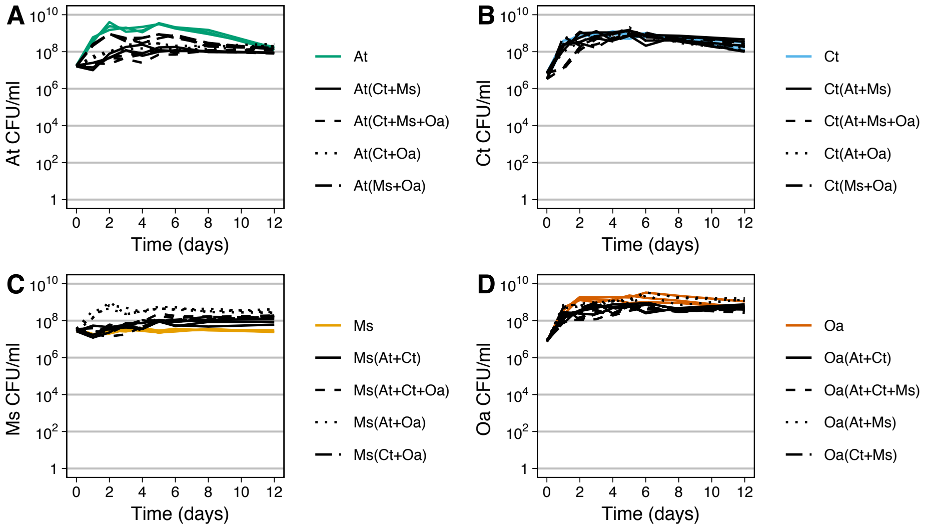

Fig. S13. Growth of three and four-species co-cultures in amino acid medium (AA). Legend explanations are as in Fig. 1.

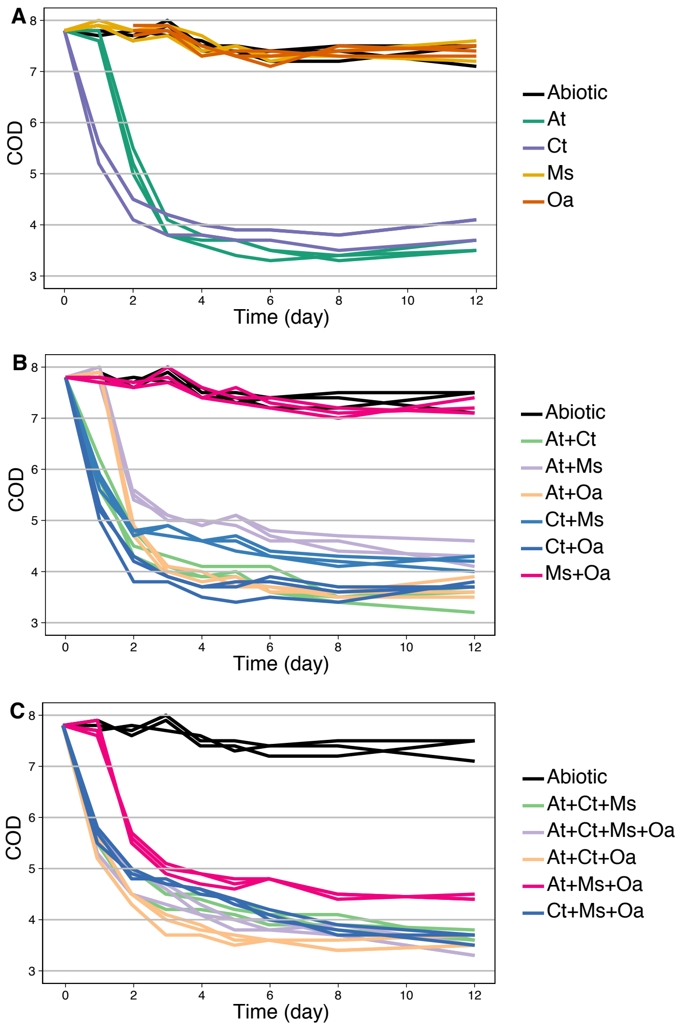

Fig. S14. Chemical Oxygen Demand (COD) over time in MWF+AA medium in (A) mono-cultures, (B) pairwise co-cultures and (C) in three- and four-species co-cultures. Each treatment was performed in triplicate. Black lines show an abiotic control treatment that was sterile. Note that these absolute COD values are not directly comparable to those in Fig. S6, since for each experiment the values in the abiotic control differ somewhat.

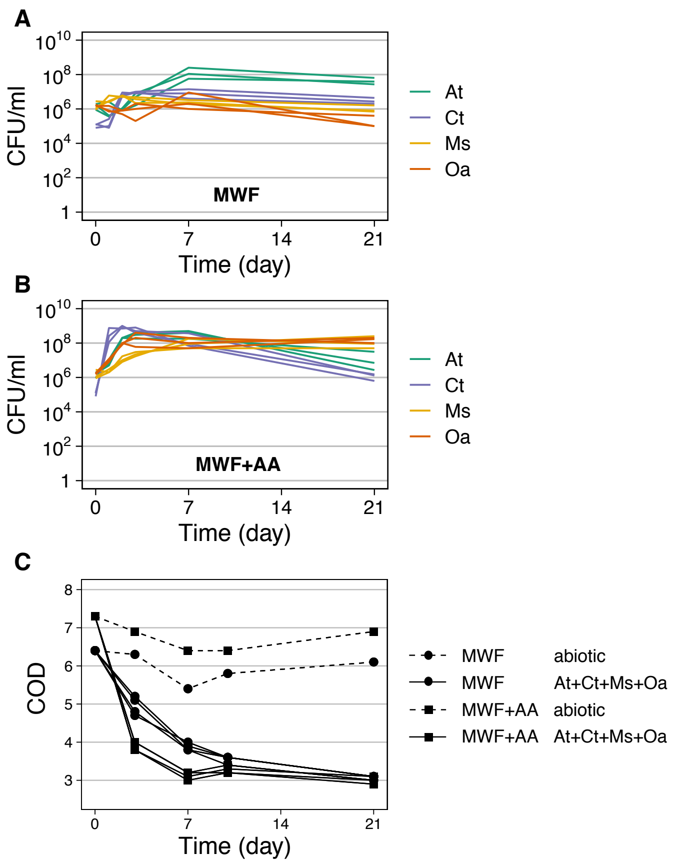

Fig. S15. CFU/ml and Chemical Oxygen Demand (COD) over time in an additional experiment where all four species were grown in MWF and in MWF+AA in parallel. (A) Growth in MWF as well as (B) in MWF+AA stabilizes after around 7 days, and matches results shown in Fig. S11 and S12. (C) COD decrease changes little after 12 days corresponding to the length of our experiment. Note that in each experiment, the starting COD of the control is somewhat different, making it difficult to compare COD values between experiments. Here, however, we can directly compare the COD decrease in MWF and in MWF+AA. These data show that AA has a COD of approximately 0.9g/L, and that the four species appear to degrade similar substrates independently of the presence of AA, which can be seen in how the COD converges to a similar value in the two treatments.

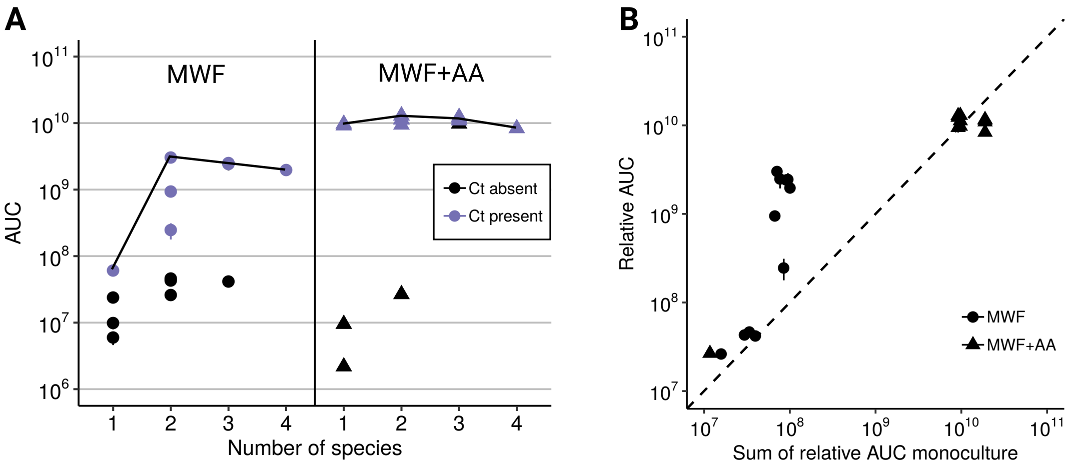

Fig. S16. Same as Fig. 5 in the main text but rather than the COD data and the AAC, here we use the total CFU/ml data and the our growth measure (AUCs) of each mono- and co-culture to observe at what species number the overall population size saturates. In (A) we ask whether the carrying capacity increases with additional species and find that in MWF it reaches its maximum at 2 species, while in MWF+AA, it is already reached at 1 species. (B) The sum of AUCs in mono-culture do not always predict the co-culture. Again, in MWF, populations are sometimes larger in co-culture than expected by the additive null model, while for MWF+AA, the null model is a good predictor for the total in co-culture. Note that the scale here is logarithmic.

Medium	Product	Concentration
MWF	K_2HPO_4	0.6g
	KH_2PO_4	0.6g
	HMB	20ml
	Castrol Hysol™ XF	500ml
	H_2O	to complete 1L
MWF+AA	K_2HPO_4	0.6g
	KH_2PO_4	0.6g
	HMB	20ml
	Castrol Hysol™ XF	500ml
	casaminoacids	0.1g
	H_2O	to complete 1L
AA	K_2HPO_4	0.6g
	KH_2PO_4	0.6g
	HMB	20ml
	casaminoacids	0.1g
	H_2O	to complete 1L
HMB	NTA (nitric triacetic acid)	10g
	$MgSO_4.7H_2O$	14.45g
	$CaCl_2.2H_2O$	3.33g
	$(NH_4)_6Mo_7O_{24}.4H_2O$	0.00974g
	$FeSO_4.7H_2O$	0.099g
	Metals44	50ml
	H_2O	to complete 1L
Metals44	$Na_2EDTA.2H_2O$	0.387g
	$ZnSO_4.7H_2O$	1.095g
	$FeSO_4.7H_2O$	0.914g
	$MnSO_4.7H_2O$	0.154g
	$CuSO_4.H_2O$	0.0392g
	$Co(NO_3)_2.6H_2O$	0.0248g
	$Na_2B_4O_7.10H_2O$	0.0177g
	H_2O	to complete 100ml

Table S1. Chemical composition of media used.

Table S2. P-values behind interactions in Fig. 3 are shown in Dataset S1.

Param	$r_{\max}$	$m_{\max}$	Y	δ	κ	f	K_N	K_T
Figure 2B	0.1	0.2	0.2	15.0	$1.0E-3$	0.1	1.5	1.0
Figure 2C, 4AB	0.1	0.15	0.2	10.0	$1.0E-2$	0.6	1.0	1.0

Table S3. These parameters were used to run the examples in Figures 2B, C and 4A, B in the main text. The parameters were tuned to show representative dynamics of the mathematical model described in Eq. (S3a)–Eq. (S3c).

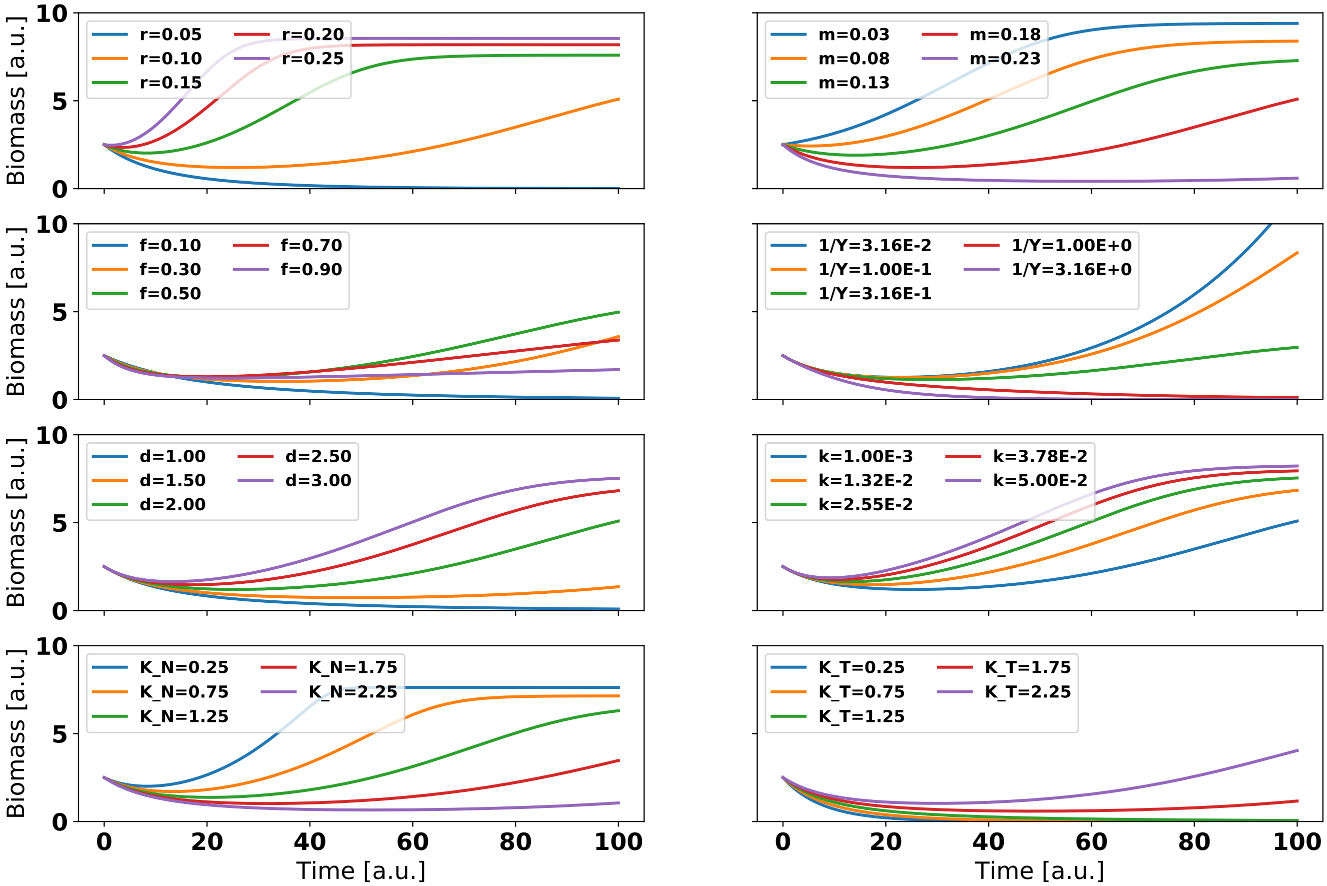

Fig. S17. Effect of parameters on simulated mono-culture biomass in the mathematical model Eq. (S3a)-Eq. (S3c). Row-wise from top left to bottom right: maximum growth rate r_{max}, maximum death rate m_{max}, degradation investment f, inverse of biomass yield Y, degradation rate δ, passive degradation rate κ, nutrient and toxin saturation constants K_N, K_T. When one parameter is changed, the rest are kept at the standard value found in Table S3.

Supplementary Note S3: Differential toxin degradation allows for density-independent facilitation

The original formulation in the main text (copied below for ease of reading) is valid for n species affected by a single toxin and sharing a single nutrient. For simplicity, in the main text we assume that the two species are identical in all parameters, while in practice they will probably vary in their mortality rates m, toxin degradation rates δ, growth rate r and biomass yield Y. Recall that the we use Monod kinetics for the growth function $\rho_i(C_N) = r_i C_N/(C_N + K_N)$, (2A) and toxin-dependent mortality $\mu_i(C_T) = m_i C_T/(C_T + K_T)$, (2B).

$$\frac{dS_i}{dt} = ((1-f_i)\rho_i(C_N) - \mu_i(C_T))S_i \quad \textbf{(S3a)}$$

$$\frac{dC_N}{dt} = -\sum_{i=1}^{n} \frac{1}{Y_i}\rho_i(C_N)S_i \quad \textbf{(S3b)}$$

$$\frac{dC_T}{dt} = -C_T \sum_{i=1}^{n} (f_i \delta_i \rho_i(C_N) + \kappa_i)S_i \quad \textbf{(S3c)}$$

Importantly, we have shown in the main text that both positive and negative interactions may occur in an environment that includes toxins, where the positive interactions relies on increasing the total population size from mono- to co-culture. If instead the total population size is kept constant by inoculating the n species co-cultures with initial abundance $\frac{1}{n}$, only negative interactions will be found, because the same resources will be divided amongst all strains. On the contrary, our experiments revealed positive interactions even when the total population size was held constant (Fig. S5).

To better capture these positive interactions when scaling the inoculum in this manner, we modified the model to include two toxins T_1 and T_2, which affect both strains equally, as shown in Eq. (S4a)–Eq. (S4e) and Fig. S18A, where T_1 is degraded by strain S_1 while S_2 degrades T_2. At intermediate nutrient concentrations, we again observe two-way positive effects of being in co-culture (Fig. S18) since the degrading member of each toxin facilitates the environment for the other by reducing the concentration of one of the two toxins.

$$\frac{dS_1}{dt} = ((1-f_1)\rho_1(C_N) - \mu_1(C_{T_1}) - \mu_2(C_{T_2}))\,S_1 \quad \textbf{(S4a)}$$

$$\frac{dS_2}{dt} = ((1-f_2)\rho_2(C_N) - \mu_1(C_{T_1}) - \mu_2(C_{T_2}))\,S_2 \quad \textbf{(S4b)}$$

$$\frac{dC_N}{dt} = -\left(\frac{1}{Y_1}\rho_1(C_N)S_1 + \frac{1}{Y_2}\rho_2(C_N)S_2\right) \quad \textbf{(S4c)}$$

$$\frac{dC_{T_1}}{dt} = -C_{T_1}(f_1\delta_1\rho_1(C_N)S_1 + \kappa_1 S_1) \quad \textbf{(S4d)}$$

$$\frac{dC_{T_2}}{dt} = -C_{T_2}(f_2\delta_2\rho_2(C_N)S_2 + \kappa_2 S_2) \quad \textbf{(S4e)}$$

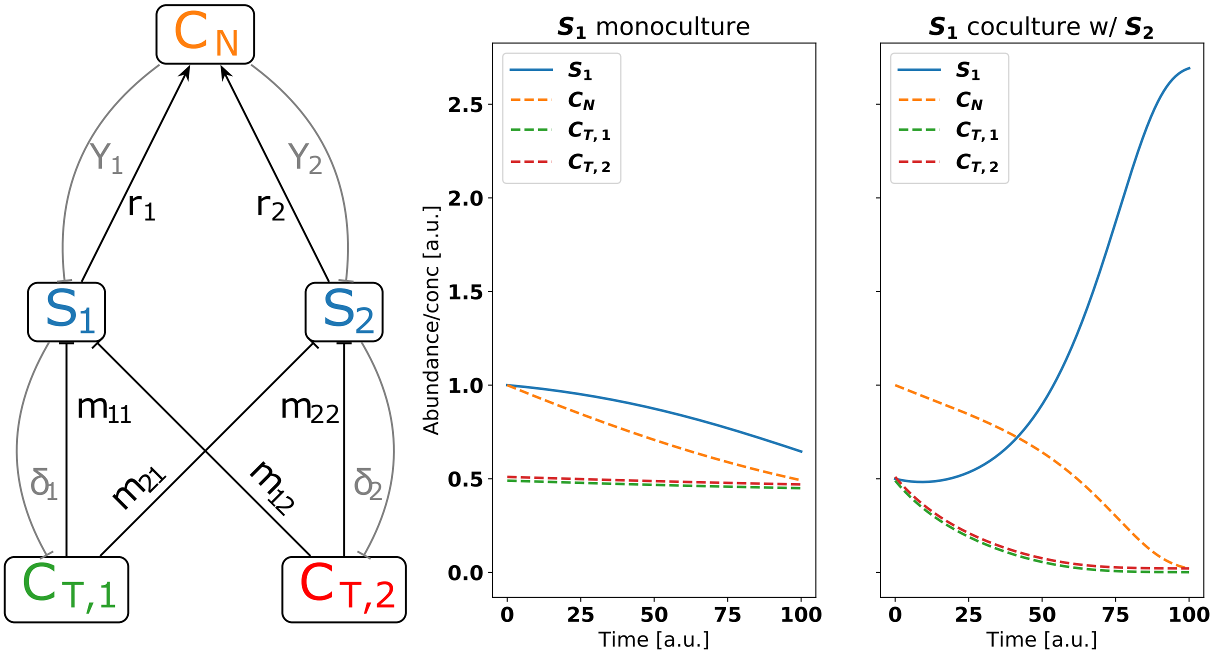

Fig. S18. (**A**) We extend the model by introducing two toxins that affect both species but can only be degraded by one species each. (**B**) The simulated biomass shows positive interactions where both species are better off in co-culture even while the total inoculated biomass is the same between mono- and co-culture. In the example run, the effect of nutrients and toxins is symmetric by the choice of equal parameters for S_1 and S_2, and the two species only differ in their degradation abilities for the two toxins.

Supplementary Note S4: Supplementary text

Facilitation is not due to inoculum doubling. In our experimental setup, the total initial inoculated population was larger in co-cultures compared to mono-cultures. If all four species detoxify and degrade the same exact compounds in MWF, positive interactions could be explained by this larger initial cell density. Alternatively, if species differ in their contribution to detoxification, positive interactions should be maintained even if we keep the initial cell density constant across treatments.

To differentiate between these possible explanations, we repeated the experiment with a constant total inoculum volume across pairwise co-cultures and mono-cultures. All species still grew significantly better in the presence of *C. testosteroni*, and *C. testosteroni* benefited from all others (Fig. S5). However, *M. saperdae* and *O. anthropi* died faster in pairwise co-cultures compared to mono-cultures if their partner was also dying. Worse growth was presumably due to halving the focal species' inoculum, rather than a real negative interaction between these species pairs. Indeed, doubling the number of cells in mono-culture showed a significant improvement for all species (F-test, df=3, all $P < 0.015$). The fact that species grow worse with a smaller inoculum supports our initial experimental design as presented in the main text, where we kept the cell numbers of each species constant across treatments. The original design allows us to avoid confounding initial population size with the presence of another species.

Taken together, the additional experiment (Fig. S5) shows that even though the starting population size of mono-cultures influences survival, the four species appear to functionally complement each other in facilitating growth and survival in MWF.
